# An Unusual Case of Extrapulmonary Hydatid Cyst Masquerading as a Mediastinal Tumor

**DOI:** 10.7759/cureus.5612

**Published:** 2019-09-10

**Authors:** Bandhul Hans, Kamesh Gupta, Kshitij Kalra, Jagdish C Suri

**Affiliations:** 1 Forensic Medicine, All India Institute of Medical Sciences, New Delhi, IND; 2 Internal Medicine, Baystate Medical Center, Springfield, USA; 3 Pulmonology, B.J. Medical College, Pune, IND; 4 Pulmonology, Vardhman Mahavir Medical College, New Delhi, IND

**Keywords:** hydatid cyst, extrapulmonary hydatid cyst, mediastinal tumor, extrahepatic hydatid cyst

## Abstract

Hydatid disease is a common disease in developing countries. The usual presentations include lung and liver cysts. Herein, we present a case of extrapulmonary, intrathoracic hydatid cyst with chest wall and spinal cord involvement, with the patient having symptoms of neurological compression and chest pain. Contrast-enhanced computed tomography (CECT) showed a large, septated, cystic mass which was eroding third, fourth and fifth ribs posteriorly, undermining the transverse process and pushing the spinal cord to the right through the intervertebral foramen. The diagnosis was confirmed by aspiration cytology. The patient was treated with albendazole as she refused surgery, which showed complete resolution of symptoms within one month.

## Introduction

Hydatid disease (echinococcosis) is caused by parasitic infection with cestode worms belonging to the genus Echinococcus. It is endemic in the Mediterranean region and in areas where livestock is raised in association with dogs. The mode of transmission is by ingesting the larval eggs. After embryos escape from eggs, they penetrate the intestinal mucosa, enter portal circulation and are carried to many organs, most commonly liver or lungs. However, cysts can also appear in other areas of the body [1-2]. Intrathoracic extrapulmonary hydatid disease constitutes 7.4% of all hydatid diseases [3]. Typical pulmonary or hepatic hydatid cysts are not challenging to diagnose. Conversely, diagnostic difficulties arise when cysts appear intrathoracically, but in extrapulmonary locations. Cysts in such sites can lead to fatal complications, such as bronchial rupture, fistulas to the pleural and pericardial cavities, and severe bleeding [4].

## Case presentation

A 22-year-old female native of South Asia presented with the history of left-sided infra-clavicular chest pain and numbness in the left forearm for the past three months. It was progressive, continuous in nature, pleuritic in character. She had no history of fever, cough, breathlessness, hemoptysis, or weight loss. She did not consume alcohol or tobacco in any form. She had no prior history of diabetes/hypertension/asthma/any other chronic medical condition.

On physical examination, her vitals were within normal limits. She had no significant lymphadenopathy. The respiratory examination revealed dullness on percussion, decreased vocal fremitus, and diminished breath sounds in left supra-scapular and interscapular regions. The rest of the physical examination was unremarkable.

**Figure 1:**
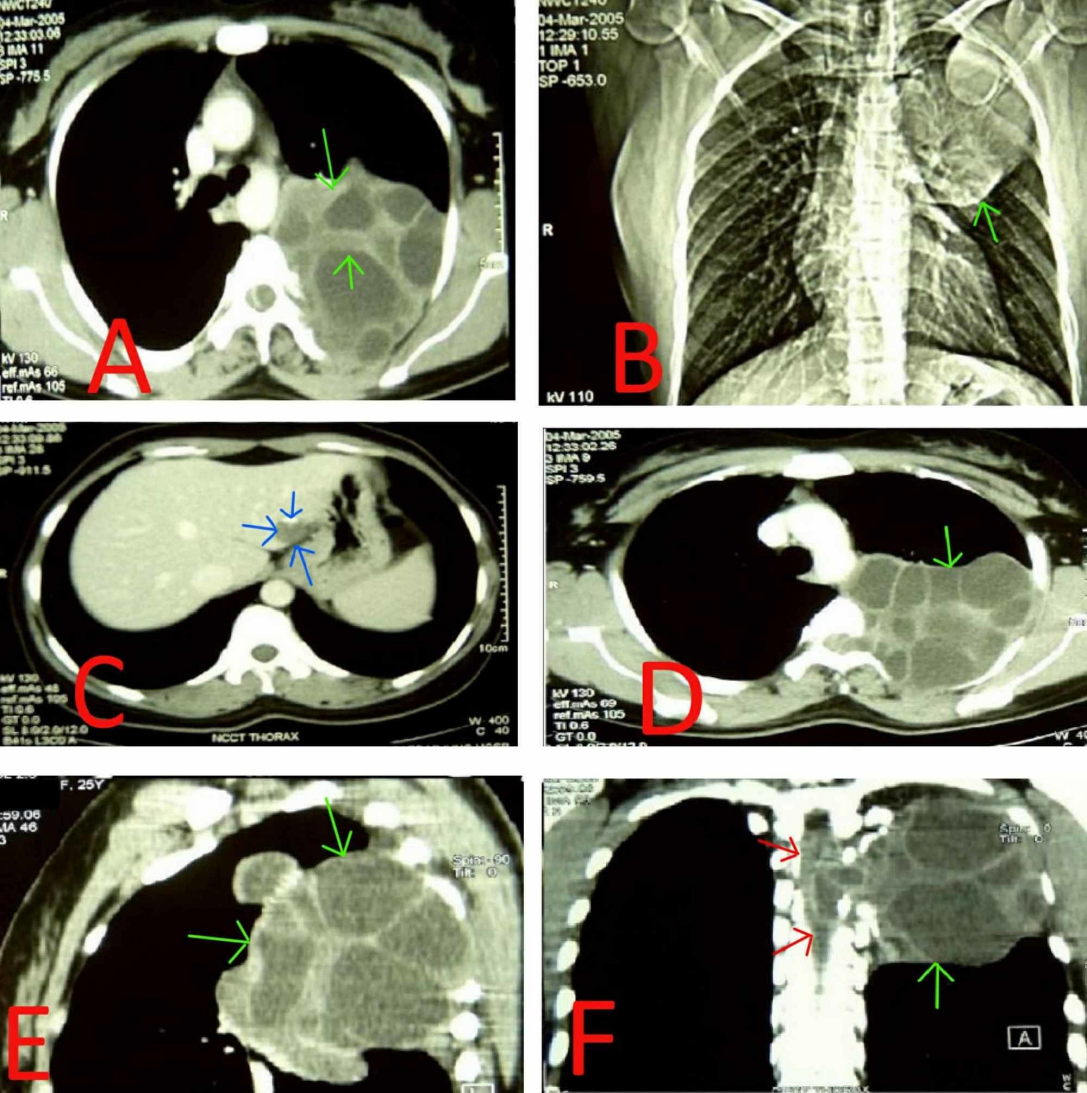
Noncontrast CT of chest and abdomen. (A) & (D) Shows a large, lobulated and septated cystic lesion (green arrows) in left para vertebral gutter. (B) Shows the superior inferior extent of the lesion, extending from just below the thoracic inlet to the level of the left atrium (green arrows). (C) Liver shows a non-enhancing, septated cystic lesion with eccentric enhancement in the medial segment of the left lobe of liver (blue arrows). Spleen, gall bladder, intra hepatic biliary radicals and kidney appear normal. (E) Shows the anterior-posterior extent of the lesion (green arrows). (F) Coronal plane view showing the lesion (green arrows) and the extension of the lesion (red arrows) in to the spinal canal hence causing neurological symptoms.

The baseline lab investigations were significant for the absence of leucocytosis; erythrocyte sedimentation rate (ESR) was normal but arterial blood gas (ABG) analysis showed hypercarbia with incomplete metabolic compensation. Pulmonary function tests (PFTs) showed a mild restrictive ventilatory defect. The chest X-ray and chest CT without and with contrasts (Figure 1) showed a large, cystic lesion in left paravertebral gutter entering the spinal cord and abutting the chest wall. Fiber-optic bronchoscopy was done in order to obtain a biopsy; however, it did not show any masses or lesions inside the bronchi amenable to biopsy. Fine needle aspiration cytology (FNAC) of the lesion was obtained by a percutaneous approach which revealed clear, watery fluid that stained positive for scolices. Serology for echinococcosis was positive by enzyme-linked immunosorbent assay (ELISA) in both blood and aspirated fluid.

Based on the imaging and the cytology, the patient was hence diagnosed as having primary intrathoracic extrapulmonary hydatid cysts with chest wall involvement and extension into the spinal canal. The differentials ruled out based on imaging and cytology included a paraspinal abscess, vertebral osteomyelitis, and metastatic tumor to the spine.

The patient was started on albendazole 400 mg tablet twice daily for 14 days. She was given two more cycles with intervening 14 days rest. Although the standard of care is cyst drainage and excision, she refused to undergo any surgical intervention. We continued to monitor her closely. One month after albendazole therapy her pain disappeared completely. At the follow-up at two months, she was asymptomatic and a repeat X-ray showed a reduction in the mass size.

## Discussion

Intrathoracic extrapulmonary hydatid cysts are rare entities. Amongst them, 55% of cysts are located in the intrapulmonary fissure, 18% within parietal pleura, 14% in the chest wall, 4.5% in the mediastinum, and 4.5% in the diaphragm [3]. The spread to this region is believed to occur through vertebral-portal venous anastomoses [5]. When an intrathoracic extrapulmonary hydatid cyst lies in the vicinity of bony structures, it may result in bone and muscle destruction [6]. Oğuzkaya et al. found only 22 (7.4%) extrapulmonary hydatid cyst cases in 14 years, with only three involving the chest wall [3]. The data for primary chest wall hydatid disease associated with spinal canal involvement is even rarer with only one published case found by a review of literature done by Kiliç et al. [6]. The early phase is usually asymptomatic. Spinal hydatid cysts are space-occupying lesions and produce signs and symptoms due to their mass effect. Most common signs and symptoms are paraparesis (62%), and back or radicular pain (55%) [6].

Diagnosis is made by imaging or serological testing. CT and MRI recognize certain details of lesions not detected by conventional radiography [3]. The serological and molecular diagnostic methods include indirect hemagglutination tests, ELISA, monoclonal antibodies (MAbs), and aspiration cytology [4].

Surgery is considered to be the most efficient option for treating spinal canal hydatid cysts [6]. The aim of surgery is to remove the entire cyst(s) without rupture. Laminectomy with simple decompression is used most frequently. Despite therapy, the disease often relapses with progressive destruction of the vertebral column and neurological deterioration. However, pre- and postoperative, albendazole is desired. The rate of recurrence of spinal cysts is 24.32%, and the incidence of paraplegia due to progressive disease is 24%-45% [5].

## Conclusions

This case highlights a hydatid cyst mimicking symptoms of an intrathoracic tumor causing neurological compressive symptoms. Index for suspicion must be high if the patient presents with a liver or lung cyst, especially if he/she is in close contact with livestock. Although extrapulmonary intrathoracic cysts are well documented, those involving spinal canal are quite rare. Resulting spinal cord compression can cause neurological symptoms. Serological testing and imaging remain the mainstay of diagnosis. Surgery remains the mainstay of treatment, but like in our case, can be treated with a long duration of albendazole therapy.
